# Molecular cloning, characterization, genomic organization and promoter analysis of the α1,6-fucosyltransferase gene (*fut8*) expressed in the rat hybridoma cell line YB2/0

**DOI:** 10.1186/1472-6750-11-1

**Published:** 2011-01-05

**Authors:** Béatrice Teylaert, Edwige Meurice, Marie Bobowski, Anne Harduin-Lepers, Christine Gaucher, Alexandre Fontayne, Sylvie Jorieux, Philippe Delannoy

**Affiliations:** 1 Laboratoire Français du Fractionnement et des Biotechnologies, Lille, France; 2 Univ. Lille Nord de France, F-59000 Lille, France; 3 USTL, UGSF, F-59650 Villeneuve d'Ascq, France; 4 CNRS, UMR 8576, F-59650 Villeneuve d'Ascq, France

## Abstract

**Background:**

The rat hybridoma cell line YB2/0 appears a good candidate for the large-scale production of low fucose recombinant mAbs due to its lower expression of *fut8 *gene than other commonly used rodent cell lines. However, important variations of the fucose content of recombinant mAbs are observed in production culture conditions. To improve our knowledge on the YB2/0 fucosylation capacity, we have cloned and characterized the rat *fut8 *gene.

**Results:**

The cDNAs encoding the rat α1,6-fucosyltransferase (FucT VIII) were cloned from YB2/0 cells by polymerase chain reaction-based and 5' RNA-Ligase-Mediated RACE methods. The cDNAs contain an open reading frame of 1728 bp encoding a 575 amino acid sequence showing 94% and 88% identity to human and pig orthologs, respectively. The recombinant protein expressed in COS-7 cells exhibits a α1,6-fucosyltransferase activity toward human asialo-agalacto-apotransferrin. The rat *fut8 *gene is located on chromosome 6 q and spans over 140 kbp. It contains 9 coding exons and four 5'-untranslated exons. FISH analysis shows a heterogeneous copy number of *fut8 *in YB2/0 nuclei with 2.8 ± 1.4 mean copy number. The YB2/0 *fut8 *gene is expressed as two main transcripts that differ in the first untranslated exon by the usage of distinct promoters and alternative splicing. Luciferase assays allow defining the minimal promoting regions governing the initiation of the two transcripts, which are differentially expressed in YB2/0 as shown by duplex Taqman QPCR analysis. Bioinformatics analysis of the minimal promoter regions upstream exons E-2 and E-3, governing the transcription of T1 and T2 transcripts, respectively, evidenced several consensus sequences for potential transcriptional repressors. Transient transfections of Rat2 cells with transcription factor expression vectors allowed identifying KLF15 as a putative repressor of T1 transcript in Rat2 cells.

**Conclusion:**

Altogether, these data contribute to a better knowledge of *fut8 *expression in YB2/0 that will be useful to better control the fucosylation of recombinant mAbs produced in these cells.

## Background

Several independent studies have clearly shown that effector functions of recombinant therapeutic IgG are directly dependent on the glycosylation of the constant region (Fc) [1-3]. Each heavy chain of IgG1 Fc fragment contains a single N-glycosylation site substituted by a biantennary complex glycan. The minimal core structure is a heptasaccharide (GlcNAc_2_Man_3_GlcNAc_2_) potentially substituted by galactose (Gal), bisecting N-acetylglucosamine (GlcNAc), sialic acid (Neu5Ac) and/or fucose (Fuc) residue α1,6-linked to the first GlcNAc attached to Asn297 of IgG heavy chains [4].

IgG Fc oligosaccharides determine the overall conformation of the Fc fragment [5] and modulate the capacity of IgG to interact with FcγR [6]. Therefore, it is clearly established that the Antibody-Dependent Cellular Cytotoxicity (ADCC) is dependent on appropriate glycosylation of the Fc region of mAbs (for review [7]). The specific role in ADCC of each monosaccharide substituting the core structure has been studied in details, showing the key role of the core fucose in cellular toxicity [8-10]. Low fucose IgG1 (10-20%) exhibit a higher ADCC activity compared to highly fucosylated IgG (80-90%) either *in vitro *[8] or *in vivo *[10].

The most widely used recombinant antibodies are produced by rodent mammalian cell lines with intrinsic fucosyltransferase activity (e.g., Chinese hamster ovary (CHO), mouse myeloma and hybridoma cell lines). Therefore, almost all licensed therapeutic antibodies developed to date are heavily fucosylated [11,12], which results in a non-optimized ADCC. Reducing the α1,6-fucose rate of IgG Fc has been a challenge over the last few years to provide maximum efficiency to recombinant mAbs.

In mammals, the GDP-L-Fuc: N-acetyl-β-D-glucosaminide α1,6-fucosyltransferase (α1,6-FucT) is the only enzyme able to catalyze the transfer of a Fuc residue in α1,6-linkage to the first GlcNAc residue of N-glycan chains [13]. GDP-fucose, the unique donor substrate of fucosyltransferases, is synthesized in the cytoplasm from GDP-mannose, via three enzymatic reactions carried out by two proteins: GDP-mannose 4,6-dehydratase (GMD) and GDP-4-keto-6-deoxymannose 3,5-epimerase, 4-reductase (FX) [14,15]. The GDP-fucose is then transported into the lumen of the Golgi apparatus by a GDP-fucose transporter (GFT) located at the Golgi membrane [16], where it serves as a substrate in the synthesis of fucosylated glycoconjugates [15,17,18]. α1,6-FucT is encoded by the *fut8 *gene and different strategies targeting *fut8 *or other fucose-related genes have been developed to reduce the fucosylation capacity of recombinant mAb producing cells. The *fut8 *gene [19], the GMD gene [20], or both [21] have been knockdown in CHO cells, generating completely non-fucosylated recombinant mAbs. *Fut8 *siRNA was also used for engineering CHO cells to upgrade effector function of produced antibodies [22] and recently, short-hairpin-RNA has been developed for the silencing of *fut8 *in CHO, resulting in an enhanced antibody immune effector function [23]. Glycosylation inhibitors were also proposed to enhance cellular toxicity of recombinant IgG [24] and lectin-affinity chromatography techniques have been applied to enrich in non-fucosylated species the produced recombinant mAbs [25].

Alternative cell lines have been tested for the reduction of core fucose on recombinant mAbs. The Lec13 mutant CHO cell line, partially deficient in GMD [26] was used to produce human IgG1 that were deficient in fucose [8]. As an alternative, the rat hybridoma cell line YB2/0 was proposed as a good candidate for the large-scale production of low fucose IgG [9]. Thus, this cell line has a very good capacity of IgG biosynthesis and its fucose transfer capacity is much lower than the other rodent cell lines commonly used [27]. However, important variations of the fucose content has been observed for the producing clones in production culture conditions [28], indicating that a better knowledge of the capacity of the YB2/0 fucosylation machinery is required for optimizing the fucosylation of recombinant mAbs at the production level. Thus, we have analyzed the fucosylation capacity of YB2/0 and we report here the molecular cloning of the rat *fut8 *cDNA, the *fut8 *gene organization including 5'-untranslated exons and transcription start-sites, the number of *fut8 *copies in YB2/0 nuclear genome and the analysis of two promoter regions (P1 and P2) governing *fut8 *transcription in these cells. In particular, we identified several potential repressor binding sequences in both minimal promoters and showed that KLF15 can repress the activity of P1 promoter region.

## Methods

### Cell culture

The rat cell line YB2/0 was obtained from American Type Culture Collection (Manassas, VA, http://www.atcc.org; catalog n° CRL-1662) and grown in EMS™ medium (optimized and patented culture medium developed by the Laboratoire Français du Fractionnement et des Biotechnologies, Lille, France) supplemented with 5% heat inactivated FCS (Invitrogen, Carlsbad, CA, USA) at 37°C under 5% CO_2_. The rat Rat2 cell line (ATCC n° CRL-1764) and COS-7 (ATCC n° CRL-1651) were cultivated in Dulbecco's modified Eagle's medium, DMEM, (BioWittaker, Lonza, Brussels, Belgium) supplemented with 10% heat-inactivated FCS and 2 mM of L-glutamine (Lonza, Brussels, Belgium) at 37°C in 5% CO_2_.

### Bio-informatics analysis

*In silico *analysis was performed with BLAST analysis of rat genomic and expressed sequence tags (EST) divisions of the NCBI databases http://www.ncbi.nlm.nih.gov/sites/entrez. Multiple sequence alignments were performed with the Clustal W program http://www.ebi.ac.uk/Tools/clustalw2/index.html. Expasy site http://www.expasy.ch was used for protein analysis. Analysis of minimal promoter regions was performed with Matinspector 2.2 http://www.genomatix.de using TRANSFAC matrices 4.0 [29] with "core similarity: 0.9" and "matrices similarity: Optimized". Filters corresponding to myeloid and lymphoid cells were used to screen specific cells transcription factors.

### RNA isolation and cDNA synthesis

YB2/0 cells were used as source of RNA. Total RNA was extracted using the Nucleospin RNA II (Macherey-Nagel, Düren, Germany) according to the protocol provided by the manufacturer. Total RNA (2 μg) was reverse-transcribed using first strand cDNA synthesis kit (Amersham Biosciences, Freiburg, Germany).

### Molecular cloning of rat α1,6 fucosyltransferase cDNA

The α1,6-fucosyltransferase open reading frame was amplified from 1 μL of YB2/0 cells cDNA with the primer pair 5F8r2: 5'-agtcgccacaggattacc-3'and 3F8r2: 5'-atctgcttagccgagatg-3' at 94°C for 2 min, followed by 30 cycles (94°C, for 30 sec; 50°C for 45 sec; 68°C for 2 min 10 sec) and an extension step of 10 min at 68°C. The PCR product was cloned into the pCR II-Blunt TOPO vector (Invitrogen, Carlsbad, CA, USA). The encoding sequence was isolated by digestion of the vector using HindIII and XbaI restriction sites. The purified fragment was subcloned into pcDNA3.1 vector (Invitrogen, Carlsbad, CA, USA) digested by HindIII and XbaI. The resultant plasmid was designed pcDNA-Fut8. COS-7 cells were transiently transfected with pcDNA-Fut8 or the empty plasmid using lipofectamine (Invitrogen, Carlsbad, CA, USA) according to the manufacturer's instructions. Cells were harvested 48 h after transfection.

### Preparation of the asialo-agalacto apotransferrin acceptor substrate

After dialysis against sodium acetate buffer (50 mM, pH 7.5), 5 mg of human apotransferrin (Sigma-Aldrich, Lyon, France) were incubated with 120 mU of *Arthrobacter ureafasciens *neuraminidase (Sigma-Aldrich, Lyon, France) and 50 mU of *Escherichia Coli *β-galactosidase (Sigma-Aldrich, Lyon, France) at 37°C for 24 h in a final volume of 10 mL. After 4 h of dialysis against water, asialo-agalacto-apotransferrin was freeze-dried. The absence of galactose and sialic acid residues was controlled by GC-MS.

### Fucosyltransferase assay

COS-7 cells, transiently transfected with pcDNA-Fut8 or the empty plasmid, were harvested by scrapping in a 4 mM EDTA solution, pelleted by low-speed centrifugation and resuspended in lysis buffer (150 mM NaCl, 50 mM Tris-HCl, pH 7.5, 1% Triton X-100), containing a cocktail of proteases and phosphatase inhibitors (Roche, Basel, Switzerland). The protein concentration of protein extracts was determined with the Micro BCA™Protein Assay Reagent kit (Thermo Fisher Scientific Inc, Rockford, IL, USA).

Enzyme assays were performed with 20 μL of protein extract (140 μg/mL), 70 mM cacodylate buffer (pH 7.2) 10 mM L-fucose, 6 μM GDP-[^14^C]-L-fucose (299 mCi/mmol, Amersham Biosciences, Pantin, France), 10 mM GDP-fucose and 100 μg asialo-agalacto human apotransferrin as acceptor substrate, in a final volume of 50 μL, at 30°C for 4 h. The reaction was stopped with 150 μL of RT water, precipitated with 1 mL of 5% phosphotungstic acid and processed for scintillation counting. The transfer of [^14^C]-fucose is expressed in cpm.

### Amplification of 5'cDNA ends by RNA-Ligase-Mediated RACE (RLM-RACE)

RLM-RACE [30] amplifications were performed using the GeneRacer kit (Invitrogen, Carlsbad, CA, USA) according to the manufacturer's instructions. Initial reverse transcription was performed with the oligo dT primer using 5 μg of total RNA. After synthesis of the first strand cDNA, PCR were performed with the GeneRacer5'Primer from the kit and a gene-specific primer 3F8Race3 5'-cttcccgtagccgtcccctggtcaa-3' at 94°C for 2 min, followed by 38 cycles (94°C, for 45 sec; 57°C for 45 sec; 68°C for 1 min 45 sec) and an extension step of 10 min at 68°C. Nested PCR were performed with the GeneRacer5'Primer Nested provided in the kit and a gene-specific primer 3F8Race4 5'-actcagccattcgcctcaagtcttc-3', using 2 μL of the first PCR amplification and the same conditions as the first PCR. PCR amplifications were performed with Taq AccuPrime (Invitrogen, Carlsbad, CA, USA) according to the provider's instructions. Nested-PCR products were size separated by Agarose gel electrophoresis, subcloned into pCR4-TOPO vector (Invitrogen, Carlsbad, CA, USA) and sequenced by Genoscreen (Lille, France).

### Reverse transcription and quantitative PCR

The expression of the transcripts 1 and 2 was analyzed by quantitative real-time PCR. 2 μg of total RNA was reverse transcribed with poly-T oligonucleotide using Affinity script QPCR cDNA Synthesis kit (Agilent technologies, Santa Clara, CA, USA) according to the protocol provided by the manufacturer. Parallel reactions without SuperScript (RT controls) were performed to assess the degree of contaminating genomic DNA.

Duplex Taqman™ QPCR and subsequent analysis were performed using the Mx-4000 Quantitative System (Stratagene, Amsterdam, the Nederlands). Primers and probes were designed using Primer Express software (Applied Biosystems, Carlsbad, CA, USA) (Table 1). The probes were MGB Taqman™ probes synthesized by Applied Biosystems. The Transcript 1 (T1) and Transcript 2 (T2) probes were FAM-labeled and the reference gene HPRT probe was VIC-labeled. PCR reactions were performed with 12.5 μL of the Quantitect Multiplex PCR kit (Qiagen, Courtaboeuf, France), 0.4 μM of each primer, 0.2 μM of HPRT Taqman™ probe, 0.2 μM of T1 or T2 Taqman™ probe and 2 μL of cDNA in a final volume of 25 μL. After 95°C for 15 min, 40 PCR cycles were performed as follows: 95°C for 1 min, 60°C for 1 min. Assays for each transcript were performed in triplicate and real-time PCR amplification was repeated 3 times. Relative amounts of mRNAs were based on standard curves prepared by a serial dilution of control YB2/0 cDNA.

**Table 1:** Probes and primer sequences used for TaqMan™ quantitative PCR experiments

*Target*	*Forward Primer* *Reverse Primer*	*Probe*
Transcript T1	5'-cgcgggctgctgttc-3' 5'-ataataatggaaaggacttgatcttgg-3'	5'-ccctggtggcgtt-3'
Transcript T2	5'-ccgcttagctcgccctcta-3' 5'-aggactagatcttggatgaaaacg-3'	5'-agtccttcggcccacg-3'
HPRT	5'-tgtacttggcttttccactttcg-3' 5'-tgcccttgactataatgagcacttc-3'	5'-atgacacaaacatgattca-3'

### FISH (Fluorescence In Situ Hybridization)

The YB2/0 cell line was grown in EMS™ medium supplemented with 5% heat inactivated FCS for 72 hours prior cell fixation and *in situ *hybridization. Primary rat lymphocytes were isolated from rat blood following standard procedures. The probes used for the FISH analysis were designed and prepared as follow: fut8 and a non relevant probe (NRP) for rat genome were designed by using respectively 3 and 2 BACs. Bacteria were amplified in LB Broth, and then BACs were purified on Anion Exchange columns (Qiagen, Courtaboeuf, France) following the manufacturer's instructions. Probes labeling was then performed using the ARES Alexa Fluor 488 Labeling kit (Invitrogen, Carlsbad, CA, USA), while the purification was done with the PureLink PCR Purification Kit (Invitrogen, Carlsbad, CA, USA). To avoid probes unspecific binding to repetitive DNA sequences, samples were precipitated with 1 μg of Rat Hybloc competitor DNA (Insight Biotechnology Limited, Wembley, UK).

The FISH assays were carried out in standard conditions. Fluorescent DNA probes and samples were denatured for 10 min at 73°C. The hybridizations were performed overnight at 37°C in a humidified chamber. The coverslides were then washed 3 times at 45°C with 50% formamide in SSC2X, one time in SSC2X before a final wash in SSC2X, 0.1% NP-40. Finally, nuclei were counterstained for 30 min at 4°C in darkness, with DAPI III/mountain medium (Abbott-France, Rungis, France) before observation under an epifluorescence microscope (Olympus, BX61) using appropriated fluorescent filters.

Raw data of the FISH analysis were compared with statistical methods by using SPSS 13.0 software. Data were analyzed under the normality test Kolmogorov-Smirnov, before applying non parametric Kruskal-Wallis and U-Mann Whitney tests. Statistical differences between samples were considered significant when p < 0.05.

### Construction of reporter plasmids for luciferase assays

Genomic DNA of YB2/0 cells were prepared with Nucleospin Extract II kit (Macherey Nagel, Düren, Germany) following manufacturer's instructions. To analyze the T1 transcript promoter region, the genomic sequence located between the -1438 bp and -411 bp upstream the ATG was amplified by PCR using the primer pair: FP1.8: 5'-cgtggcaagcttccctaacgcccccttacccg-3' and FP1.7: 5'-ctccctggtacctccgtactcaataaacttccgcc-3' introducing HindIII and KpnI restriction sites (underlined in the oligonucleotide sequences), respectively. The PCR product was subcloned into pGL3-basic vector (pGL3b, Promega, Madison, USA) upstream of the *firefly *luciferase gene at HindIII/KpnI sites. The resulting plasmid was designed pGL3(-1438/-411). This PCR fragment was submitted to 5' and 3' deletions using natural restriction enzyme sites and cloned in pGL3b to generate various vectors (Table 2).

**Table 2:** Reporter plasmids for T1 transcript luciferase assays.

*Digestion*	*Position of the fragment*	*Vector name*
ApaI, HindIII	-1205/-411	pGL3(-1205/-411)
BseRI, HindIII	-1004/-411	pGL3(-1004/-411)
NheI, HindIII	-892/-411	pGL3(-892/-411)
MseI, HindIII	-598/-411	pGL3(-598/-411)
KpnI, PvuI	-1438/-451	pGL3(-1438/-451)
KpnI, PvuI	-1205/-451	pGL3(-1205/-451)
NheI, PvuI	-892/-451	pGL3(-892/-451)
KpnI, MseI	-1438/-598	pGL3(-1438/-598)
KpnI, MseI	-1205/-598	pGL3(-1205/-598)
NheI, MseI	-892/-598	pGL3(-892/-598)

To analyze the T2 transcript promoter region, the genomic region of 1,000 bp upstream exon E-3 was amplified from YB2/0 cells genomic DNA using primers FKO11 5'-ccgggctagcacattccacccctgactcctaa-3' and FKO12 5'-ccggctcgaggtttcctacccgctcgcactcg-3' creating NheI and XhoI restriction sites (underlined in the oligonucleotide sequences), respectively. The amplified fragment was subcloned into pGL3b. The plasmid was called pGL3(-1399/-404). Following the same strategy, nested PCR fragments were subcloned into pGL3b upstream the *firefly *luciferase gene at NheI/XhoI sites (Table 3). The different constructions were sequenced to ensure the absence of mutations.

**Table 3:** Primers used for the construction of plasmids for T2 transcript luciferase assays.

*Forward primer*	*Reverse primer*	*Vector name*
FKO13-NheI 5'-ccgggctagcgtttatttccaacagagtacacgactc-3'	FKO12-XhoI 5'-ccggctcgaggtttcctacccgctcgcactcg-3'	pGL3(-1233/-404)
FKO15-NheI 5'-ccgggctagccaggtgaggagctgctgaaggcacagac-3'	FKO12-XhoI 5'-ccggctcgaggtttcctacccgctcgcactcg-3'	pGL3(-998/-404)
FKO17-NheI 5'-ccgggctagcgtcttgagggcagcttttctactcagc-3'	FKO12-XhoI 5'-ccggctcgaggtttcctacccgctcgcactcg-3'	pGL3(-720/-404)
FKO11-NheI 5'-ccgggctagcacattccacccctgactcctaagaccaac-3'	FKO14-XhoI 5'-ccggctcgaggtcgccggccgggtgccaccggggccaatc-3'	pGL3(-1399/-537)
FKO13-NheI 5'-ccgggctagcgtttatttccaacagagtacacgactc-3'	FKO14-XhoI 5'-ccggctcgaggtcgccggccgggtgccaccggggccaatc-3'	pGL3(-1233/-537)
FKO15-NheI 5'-ccgggctagccaggtgaggagctgctgaaggcacagac-3'	FKO14-XhoI 5'-ccggctcgaggtcgccggccgggtgccaccggggccaatc-3'	pGL3(-998/-537)
FKO17-NheI 5'-ccgggctagcgtcttgagggcagcttttctactcagc-3'	FKO14-XhoI 5'-ccggctcgaggtcgccggccgggtgccaccggggccaatc-3'	pGL3(-720/-537)

### Transient transfections and luciferase assay

Rat2 cells were transiently transfected using lipofectamine (Invitrogen, Carlsbad, CA, USA) according to the manufacturer's instructions, using 1 μg/mL of pGL3b construction and 10 ng/mL of control *Renilla *plasmid in OptiMEM medium (Invitrogen, Carlsbad, CA, USA). After 6 h, the medium was changed for fresh cell culture medium and incubated for 48 h at 37°C in 5% CO_2_. Cells were then washed one time with PBS, lysed in 150 μL of Passive Lysis Buffer (PLB, Dual Luciferase Reporter Assay System, Promega, Madison, USA) and 20 μL of lysate were used for luciferase Reporter Assay System. Luminescence was measured with the Centro luminometer (Berthold Technologies, Bad Wildbad, Germany).

The YB2/0 cells were transiently transfected with Cell line Nucleofector^® ^kitV (Amaxa Lonza, Verviers, Belgium) according to manufacturer's instructions. 2 × 10^6 ^cell pellets were resuspended in 100 μL of Nucleofector^® ^kitV solution. The cell suspensions were mixed with 2 μg of pGL3b construction and 20 ng of control *Renilla *plasmid. The mixtures were transferred into an Amaxa cuvette and submitted to nucleofection using the T-020 program. Cells were plated on 6-well plates with 2 mL of culture medium and incubated for 24 h at 37°C in 5% CO_2_. Cells were then centrifuged 5 min at 1,200 rpm, washed one time with PBS and processed for luciferase reporter assay as described for Rat2.

The pcDNA3.1 expression vector encoding the human KLF15 transcription factor (pcDNA-hKLF15) was kindly provided by Dr. Otteson, College of Optometry, University of Houston, USA. For transfection of Rat2 cells, 25 to 500 ng/mL of pcDNA-hKLF15 vector were added to the lipofection mix.

### Nuclear and cytoplasm protein extraction, Western blotting analysis

48 hours after transfection with KLF15 vector, Rat2 cells were lysed on ice in a hypotonic buffer (Hepes 10 mM, MgCl_2 _1,5 mM, KCl 10 mM, pH 7.9) supplemented with 0.125% NP-40 and protease cocktail inhibitors (Roche, Meylan, France). The lysate was centrifuged for 5 min at 10,000 g and the cytosolic fraction was obtained in the supernatant. The pellet corresponding to the nuclear fraction was lysed with hypertonic buffer (Hepes 20 mM, MgCl_2 _1.5 mM, EDTA 0.2 mM, NaCl 0.5 M, glycerol 25%, pH 7.9) supplemented with protease cocktail inhibitors on ice during two hours. The protein concentration of both fractions was determined with the Micro BCA™Protein Assay Reagent kit (Pierce, Rockford, IL, USA). 20 μg of total proteins were boiled for 10 min in reducing Laemmli sample buffer and resolved by SDS-PAGE on 8% mini-gels (Bio-Rad, Richmond, USA). After transfer onto a nitrocellulose membrane (80 mA overnight), blocking was performed using TBS (Tris Buffer Saline) containing 0.05% Tween 20 and 5% (w/v) non-fat dried milk for one hour at room temperature (RT). Incubations with 2.5 μg/mL anti-KLF15 mAb (AbCam, Cambridge, UK) or 0.04 μg/mL anti-actin mAb (Santa Cruz Biotechnology Inc., Europe) were performed one hour at RT in TBS, 0.05% Tween 20 and 5% (w/v) non-fat dried milk. After washing, membranes were incubated for 1 h at RT with 1/15,000 dilution in TBS, 0.05% Tween 20 and 5% (w/v) non-fat dried milk of horseradish peroxidase conjugated anti-goat IgG for KLF15 or anti-rabbit IgG for actin. Membranes were finally washed three times for 10 min in TBS, 0.05% Tween 20 and detection was achieved using enhanced chemiluminescence (ECL+^® ^advanceWestern blotting detection reagents, Amersham Biosciences, Little Chalfont, Buks, U.K.).

### Electrophoretic mobility shift assays (EMSA)

Biotin 5' end-labeled (-892/-451) promoter DNA probe was obtained by PCR from pGL3(-892/-451) plasmid using biotin 5' end-labeled primers (forward P1 min 5'-gctagcgccggcccgaggct-3', reverse P1 min 5'-cgatcggcgccgtcccccgt-3'). Rat2 cells nuclear extract was prepared as previously described. EMSA was performed with a Lightshift^® ^Chemiluminescent EMSA kit (Thermo Scientific, Waltham, USA) as follows. Nuclear extract (3 μg protein) was incubated for 20 min at room temperature in 1 × binding buffer, 2.5% glycerol, 5 mM MgCl_2_, 50 ng/μL Poly(dI-dC), 0.05% NP-40 and 20 fmol of biotin-labeled probe, in a final volume of 20 μL. 4 pmol of unlabeled (-892/-451) promoter DNA were added to the binding reaction mixture for competition studies. For supershift analysis, 1 μg of anti-KLF15 mAb (AbCam, Cambridge, UK) was pre-incubated with nuclear extract for 1 h at 4°C prior to the binding reaction. Reaction products were separated by electrophoresis in a 4% polyacrylamide gel (29:1, acrylamide/N,N'-methylene bisacrylmamide) in 0.5 × TBE. The protein-DNA complexes were then transferred onto a positively charged nylon membrane (Hybond-N+, Amersham Pharmacia Biotech, Uppsala, Sweden) and detected by chemiluminescence.

## Results

### Isolation and characterization of the rat α1,6 fucosyltransferase cDNA

**Figure 1:**
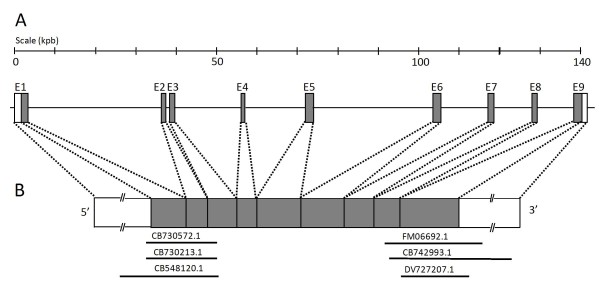
**Genomic organization of rat *fut8 *gene**. **(A) **The rat *fut8 *gene ORF is located on chromosome 6, and contains 9 exons (labeled E1-E9), spanning 140 kbp. **(B) **Schematic representation of rat *fut8 *gene mRNA. The grey boxes represent the open reading frame and the open boxes the 5'- and 3'-unttranslated regions, respectively. The black lines represent the rat EST identified in the public databases.

In order to clone the rat *fut8 *cDNA, the putative rat mRNA sequence of 1728 pb (NM_001002289.1) provided by the NCBI genome annotation division http://www.ncbi.nlm.nih.gov/sites/entrez was used as a probe for BLAST analysis of rat genomic and EST divisions of NCBI databases. The coding sequence of rat α1,6 fucosyltransferase was localized on chromosome 6, divided into nine exons and spanning over 140 kb (Figure 1A). Homologous EST sequences obtained were conceptually translated and partially overlapping sequences were assembled to build a larger open reading frame (ORF) (Figure 1B). Three ESTs CB548120.1, CB 730213.1 and CB730572.1 correspond to the 5'-region of the *fut8 *gene, and the sequences FM069692.1, DV727207.1 and CB742993.1 correspond to the 3'-region. These sequences were used to generate oligonucleotide primers located in the 5'- and 3'-untranslated regions for PCR cloning. A unique 1942 pb PCR fragment was amplified from YB2/0 cells cDNA library, cloned into the pCR II-Blunt TOPO vector and entirely sequenced.

**Figure 2:**
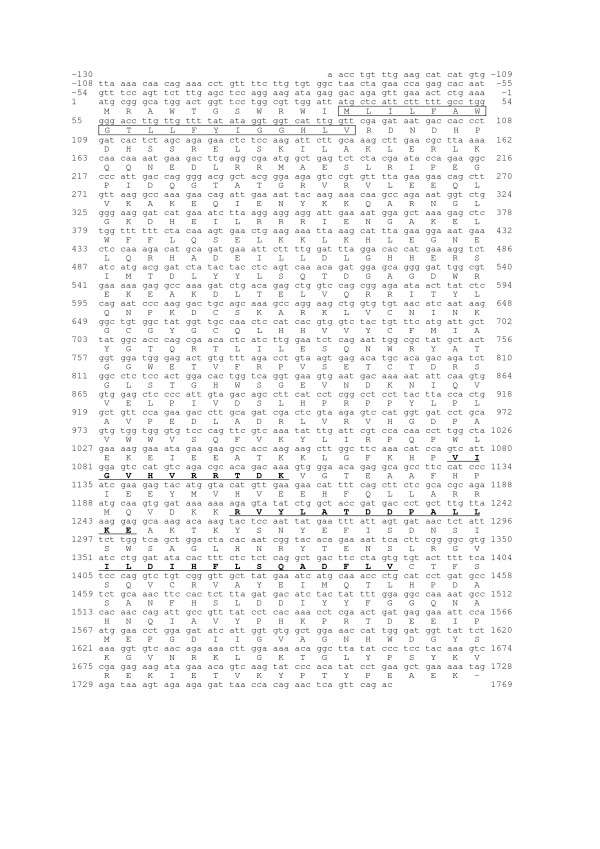
**Rat *fut8 *cDNA sequence**. Numbering of the cDNA begins with the initiation codon. The amino acid sequence is shown in single-letter code. The putative 19 amino acid N-terminal transmembrane domain is boxed. The three α1,6-fucosyltransferase specific motifs (motifs I, II & III) are underlined. The open boxes mark the position of differences with the provisional sequence.

The amplified sequence contains an ORF of 1725 bp, showing 4 nucleotide changes (at +340 (a/c), +418 (t/c), +891 (t/c) and 1313 (c/t) from ATG) compared to the putative rat *fut8 *ORF sequence (NM_001002289.1), which were also found, at least for two of them, in EST sequences of the NCBI databases (125463675/102469807, 1660740078/8735204) and reflect a silent allelic polymorphism in rat *fut8 *gene without change in the amino-acid sequence of the polypeptide. The nucleotide and deduced amino acid sequences of the full-length cDNA obtained are shown in Figure 2. The position of the initiation codon was estimated according to the Kozak consensus sequence [31]. Hydropathy analysis of the polypeptide indicated the presence of a hydrophobic sequence of 19 amino acids in the NH_2_-terminal region, corresponding to the transmembrane domain characteristic of type II Golgi glycosyltransferases. The predicted protein consisted of 575 amino acids, containing the three specific motifs of α1,6-fucosyltranferase in the catalytic domain (Figure 2). Comparison of the amino acid sequence with those of the human and pig orthologs, shows 94% and 88% identity, respectively.

In order to conclusively confirm that the YB2/0 cloned *fut8 *cDNA encodes an active α1,6-fucosyltransferase, the cDNA was inserted in pcDNA3.1 expression vector. The construction was transiently transfected into COS-7 cells. 72 h after transfection, the α1,6-fucosyltransferase activity was measured in cell extracts using asialo-agalacto apotransferrin as acceptor substrate for *in vitro *assays. The α1,6-fucosyltransferase activity was 4 times higher (2135 +/- 318 cpm) in pcDNA-Fut8 transfected COS-7 cell extracts than in the control cells (500 +/- 200 cpm), confirming that the cloned cDNA encodes an active enzyme.

### Fluorescence In Situ Hybridization

**Figure 3:**
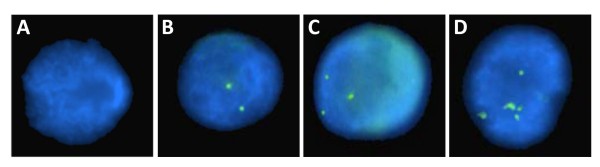
***Fut8 *copy number in interphase nuclei of YB2/0**. Picture A shows nucleus hybridized with a non relevant probe. Pictures B, C and D show YB2/0 nuclei with 2, 3 and 5 copies of *fut8*, respectively.

Copy number of *fut8 *gene was counted on at least 50 interphase nuclei of YB2/0 cell line and appropriated controls. Results are summarized as percentage and mean copy number in Table 4 and representative pictures of the different samples are shown in Figure 3. It appears that the rat myeloma cell line YB2/0 has a mean copy number of *fut8 *gene of 2.8 ± 1.4 whereas the rat primary lymphocyte has only 2.3 ± 0.8 of the same gene and the negative control with the NRP shows no hybridization. These differences are statistically significant with p < 0.05.

**Table 4:** Number of copies of fut8 gene in YB2/0 interphase nuclei.

			Number of copies (% of cells)					
				
Sample	Type	Probe	0	1	2	3	4 or more	Mean ± SD
YB2/0	Sample	Rat fut8	6.5	ND^b^	8	60	25.5	2.8 ± 1.4
PRL^a^	Positive control	Rat fut8	ND	10	64	13	13	2.3 ± 0.8
YB2/0	Negative control	NRP^c^	100	ND	ND	ND	ND	

The number of copies of *fut8 *gene observed by FISH is expressed as a percentage of cells.

^a^: primary rat lymphocyte.

^b^: not detected

^c^: non relevant probe

### Identification of rat *fut8 *transcription start sites

**Figure 4:**
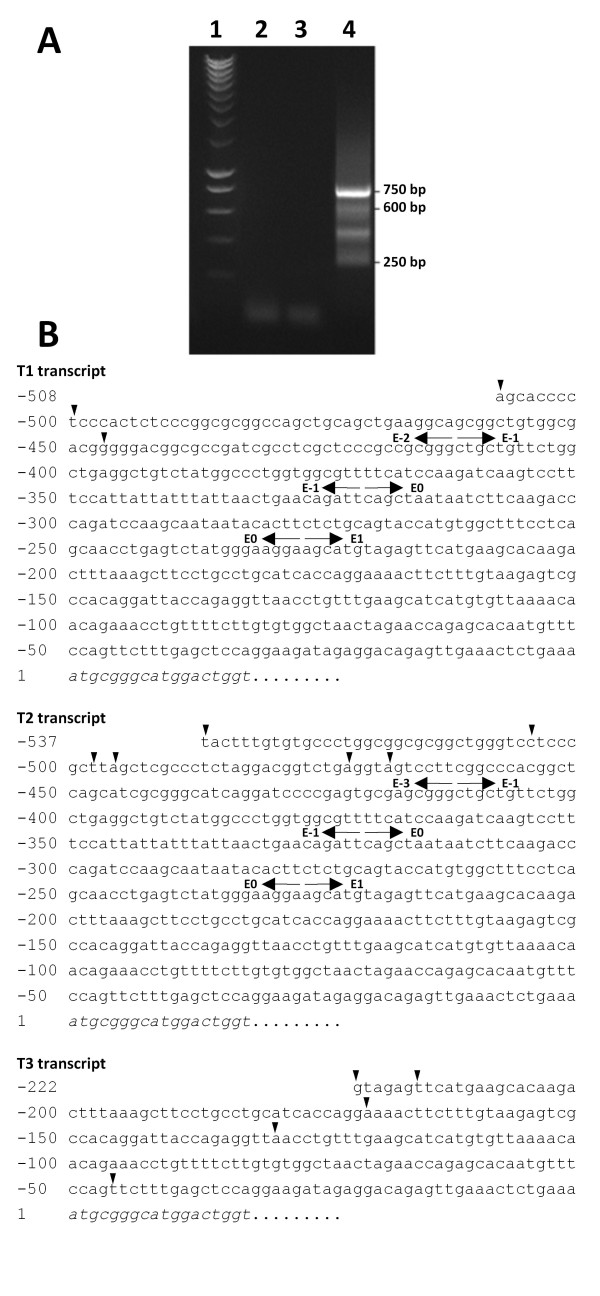
**RLM-RACE analysis of *fut8 *transcripts**. **(A) **The nested PCR products obtained by RLM-RACE of *fut8 *transcripts were analyzed on 1.2% Agarose gel. **1**: molecular ladder; **2**: negative control of amplification; **3**: PCR negative control; **4**: nested PCR products. **(B) **Nucleotide sequence of 5'-untranslated regions of T1 [EMBL: FN668936], T2 [EMBL: FN668937] and T3 [EMBL: FN668938] transcripts. Numbering begins with the initiation codon. The beginning of the coding sequence is indicated in italics. Arrowheads indicate the different transcription start sites identified by RLM-RACE. The exon limits are indicated with back-to-back arrows.

**Figure 5:**
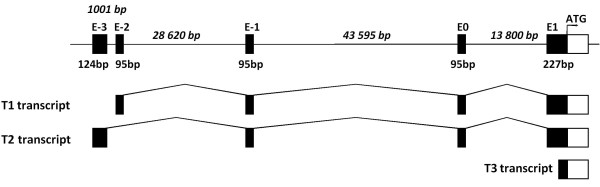
**Schematic representation of the 5'-untranslated region of *fut8 *gene and of the three transcript isoforms identified by RLM-RACE5'**. The size of introns is indicated in italics. The size of exons corresponds to the largest identified sequences.

To determine the transcriptional start sites (TSS) of rat *fut8 *gene, the mRNA 5'-ends were amplified by RLM-RACE. To ascertain to only amplify full-length transcripts, we first selected full-length capped mRNA from total RNA extracted from YB2/0 cells. For that purpose, total RNA was first treated with calf intestine alkaline phosphatase for total dephosphorylation of uncapped RNAs, thereafter the full-length mRNA was uncapped with tobacco acid pyrophosphatase. The geneRacer RNA oligonucleotide was then ligated to the 5'-phosphate of the uncapped mRNA using T4 RNA ligase. Reverse transcription was performed with the GeneRacer oligodT primer, followed by a first PCR using the forward GeneRacer5'Primer provided and the reverse gene-specific primer 3F8Race3 located at the 5'-end of exon E2. Nested PCR was performed with the forward GeneRacer5'Primer Nested provided and the reverse gene-specific primer 3F8Race4 located into the first coding exon E1. As shown in Figure 4A, which is representative of at least three independent amplifications, four bands from 250 to 750 bp were obtained. The PCR products were subcloned, sequenced and BLAST-aligned http://www.ncbi.nlm.nih.gov/BLAST/ against the rat genome. As expected, the amplified products were assigned to rat *fut8 *transcripts (NW_047761.1, NW_047771.1) excepted for the 450 bp band, which was not specific. Three different 5'-ends of *fut8 *mRNA were identified, testifying the existence of three transcript isoforms. The T1 transcript [EMBL: FN668936] corresponding to the 600 bp amplified fragment, is extended by 508 bp from the ATG and composed of 4 exons named E-2, E-1, E0 and E1 as shown in Figure 4B. Twenty different clones from three independent RLM-RACE were sequenced, defining 3 TSS for T1, at -508, -500 and -447 bp from the ATG (Figure 4B). The T2 transcript [EMBL: FN668937], corresponding to the 750 bp amplified product, is extended by about 537 bp and composed, as well, of the exons E-1, E0 and E1 but the exon E-2 is substituted by exon E-3. The sequencing of 28 clones allowed the identification of six TSS at position -537, -505, -498, -496, -473 and -469 bp from the ATG (Figure 4B). Finally, the T3 transcript [EMBL: FN668938] was the smallest one and corresponded to the 250 bp product. The T3 transcript 5'-untranslated region was only composed of the 3'-end of exon E1, with 5 TSS at position -222, -216, -171, -129 and -46 from the ATG (Figure 4B). A schematic representation of the 5'-untranslated region of *fut8 *gene and of the three transcript isoforms identified by RLM-RACEis shown in Figure 5. Similar results were obtained for Rat2 cells and rat liver RNA preparations, excepted for T3 transcript, the presence of which was not confirmed in both cases.

### Quantification of transcripts in cultured cells by duplex Taqman™ QPCR

**Figure 6:**
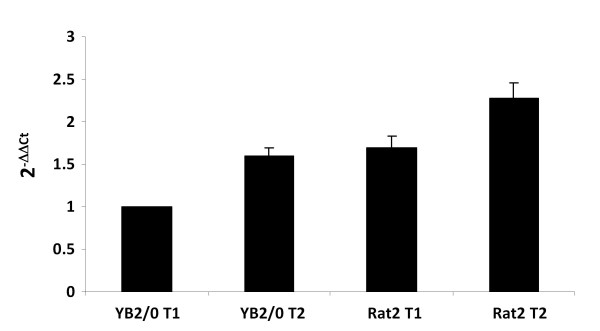
**Quantification of T1 and T2 transcripts expression in YB2/0 and Rat2 cells by duplex Taqman™ QPCR**. Results were normalized to the expression of HPRT mRNA and expressed relative to T1 mRNA expression level in YB2/0. The quantification was performed by the method described by Livak and Schmittgen [53].

The relative expression of *fut8 *specific transcripts was determined by duplex Taqman™ QPCR using *HPRT *as a normalizing gene. For T1 transcript, a forward primer hybridizing within E-2 and a reverse primer that hybridizes within the exon E-1/E-2 junction of *fut8 *cDNA sequence were designed (Table 1). For the T2 transcript, the primers pair was designed hybridizing within E-3 sequence for the forward primer and in the exon E-1 for the reverse primer. Taqman™ probes were designed within exon E-1 and E-3, respectively. For *HPRT*, primers and probe were designed to hybridize within exon 7/exon 8 sequences of the coding region (Table 1). The relative expression of specific transcripts was determined for YB2/0 and Rat2 cell lines. As shown in Figure 6, the expression of T2 transcript was 1.6-fold higher than the expression of T1. This pattern of expression was similar in both cell lines (Figure 6).

The direct quantification of T3 transcript expression was not possible because the totality of the 5'-UTR sequence is common to the sequences of the T1 and T2 transcripts. Moreover, the quantification of the total transcripts of *fut8 *using primers designed in E-1 and E0 exons didn't allow determining the expression level of T3. Nevertheless, the T3 transcript appeared to be weakly expressed in both cell lines compared to T1 and T2.

### Promoter activity of the 5'-upstream region of T1 and T2

**Figure 7:**
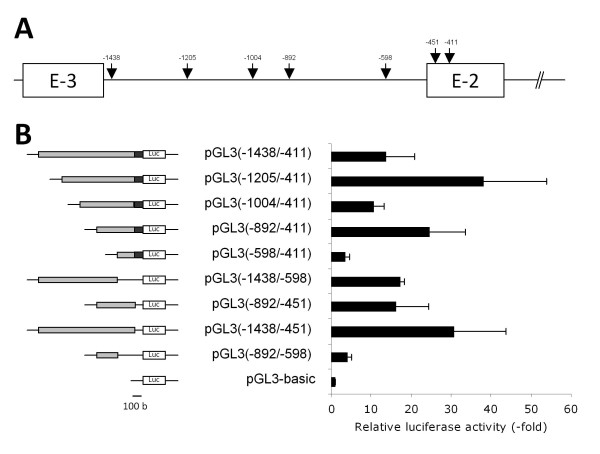
**Luciferase assays after transfections in Rat2 cells of different plasmid constructs for T1 transcription analysis**. **(A) **Location on the genomic sequence of the restriction sites used to generate the different deletions of the intronic region between exons E-2 and E-3. Numbering begins with the initiation codon. **(B) **On the left, a schematic representation of the different constructs inserted in pGL3b upstream of the luciferase gene. The gray boxes represent the different deletions of the 1 kbp intronic region between exons E-2 and E-3. The black box indicates the 5'-end of E-2. Luc indicates the *Firefly *luciferase coding sequence and the lines indicate the missing sequences. On the right: the results of luciferase assays. Transfection efficiencies were normalized with the co-transfected plasmid expressing *Renilla *luciferase and luciferase activities are expressed compared to pGL3b activity. The data are means +/- S.D. of *n *> = 3 experiments.

To determine the minimal promoter region of the T1 transcript, the 1 kb genomic sequence between E-3 and E-2 was subcloned in the pGL3b upstream the luciferase gene and named pGL3(-1438/-411). This plasmid and the 5'- or 3'-deleted constructions pGL3(-1438/-598), pGL3(-1438/-451), pGL3(-598/-411), pGL3(-892/-411), pGL3(-892/-451), pGL3(-892/-598), pGL3(-1004/-411) and pGL3(-1205/-411), were transfected into YB2/0 and Rat2 cells for luciferase assays. Unfortunately, the luminescence intensity measured in YB2/0 for these different constructions was too weak to allow determining the promoter activity of this region in this cell line. The results obtained for Rat2 are presented in Figure 7, and show a 13-fold increase of luciferase activity for the full length plasmid pGL3(-1438/-411) compared to pGL3b, whereas the luciferase activity for pGL3(-1205/-411) and pGL3(-1438/-451) constructs shows a 38- and 30-fold increased activity, respectively. In parallel, luciferase activity for the pGL3(-892/-598) and pGL3(-598/-411) plasmids show only a 3- and 4-fold increased activity compared to pGL3b. These data suggest the existence of a minimal promoter region [EMBL: FN668939] (named P1) within the sequence -892/-451, including positive and negative regulation regions in -892/-598 and 598/-451, respectively. In addition, the -1205/-1004 and -451/-411 sequences include positive regulation regions, whereas -1104/-892 sequence includes negative regulation regions.

**Figure 8:**
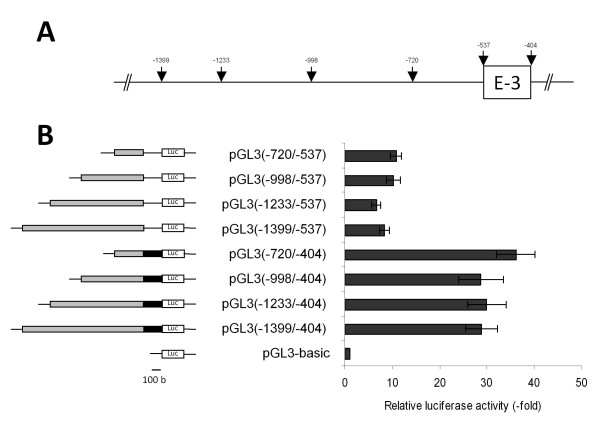
**Luciferase assays after transfections in Rat2 cells of different plasmid constructs for T2 transcription analysis**. **(A) **Location on the genomic sequence of the restriction sites used to generate the different deletions of the intronic region upstream exon E-3. Numbering begins with the initiation codon. **(B) **On the left, a schematic representation of the different constructs inserted in pGL3b upstream of the luciferase gene. The gray boxes represent the different deletions of the intronic region upstream exon E-3. The black box indicates the exon E-3. Luc indicates the *Firefly *luciferase coding sequence and the lines indicate the missing sequences. On the right: the results of luciferase assays. Transfection efficiencies were normalized with the co-transfected plasmid expressing *Renilla *luciferase and luciferase activities are expressed compared to pGL3b activity. The data are means +/- S.D. of *n *> = 3 experiments.

We also analyzed the 1 kb region located upstream the E-3 exon with or without E-3 sequence to determine the minimal promoter region controlling the transcription of T2 transcript. The full length region with the E-3 region was PCR amplified and subcloned in the pGL3b upstream the luciferase gene and named pGL3(-1399/-404). This plasmid, or 5'- or 3'-deleted constructions were transiently transfected in Rat2 cells. All the constructions increase the luciferase activity compared to pGL3b. The results presented in Figure 8 show a 7- to 11-fold increase of luciferase activity for the constructions without the E-3 exon, whereas the luciferase activity for constructs with E-3 exon shows a 28- and 36-fold increased activity. No significant differences in luciferase activity were observed between the pGL3(-1399/-537), pGL3(-998/-537) and pGL3(-720/-537) constructions. These results suggest the existence of a minimal promoter region [EMBL: FN668940] (named P2) within the sequence -720/-537 upstream the ATG and a positive regulation region in the exon E-3.

### Bioinformatics analysis of P1 and P2 minimal promoter regions

**Figure 9:**
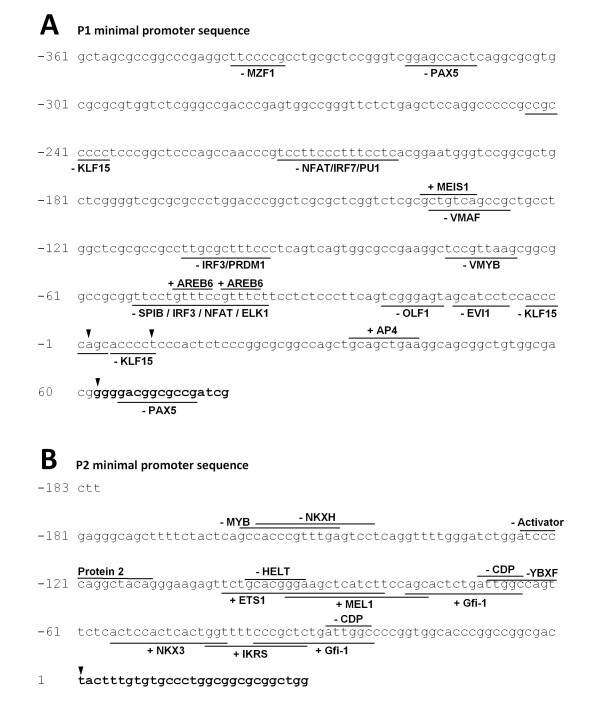
**In silico analysis of the minimal promoter regions of T1 and T2 transcripts**. **(A) **The 439 bp sequence [EMBL: FN668939] upstream the TSS (arrowheads) of the shortest T1 transcript were analyzed with Matinspector 2.2 software. Potential transcription factor binding sites are underlined with black lanes, below the sequence when the binding site is on the -strand and above when the binding site is on the + strand. Nucleotides are numbered with the nucleotide +1 as the first TSS. (**B**) The 183 bp sequence [EMBL: FN668940] defined as the minimal promoter region upstream the T2 transcript was analyzed in the same way.

The putative minimal promoter regions upstream exons E-2 and E-3 were analyzed with Matinspector 2.2 http://www.genomatix.de using TRANSAC matrices 4.0 [29]. Lymphoid and myeloid cell-specific filters were applied to sort the identified transcription factors consensus sequences and core similarity was fixed at 95%. A particular attention was put on consensus sequences for transcription factors with repressor activity, potentially usable for the control of fucosylation in YB2/0. Results of these bioinformatics analysis are shown in Figure 9. In both cases, the analyses did not reveal any canonical TATA or CAAT boxes, but several putative binding sites for general transcription factors and for transcription factors with repressor activity. For the P1 minimal promoter region (-892/-451) controlling the transcription of T1 transcript, consensus sequences for the putative repressors MZF1 (Myeloid Zinc Finger 1), PAX5 (Paired Box 5), KLF15 (Krüppel-like Factor 15), IRF3 (Interferon Regulatory Factor 3) and PRDM1 (Positive Regulatory Domain containing 1) were identified (Figure 9). For the P2 minimal promoter region, we identified potential repressor binding sites for HELT (Hey like transcriptional repressor), CDP (CCAAT displacement protein), MEL1 (MDS1/EVI1-like gene 1), NKX3.1 (androgen-regulated homeobox protein), IKRS (Ikaros) and the zinc-finger transcription factor Gfi-1.

### Effect of KLF15 over-expression on the P1 minimal promoter activity

**Figure 10:**
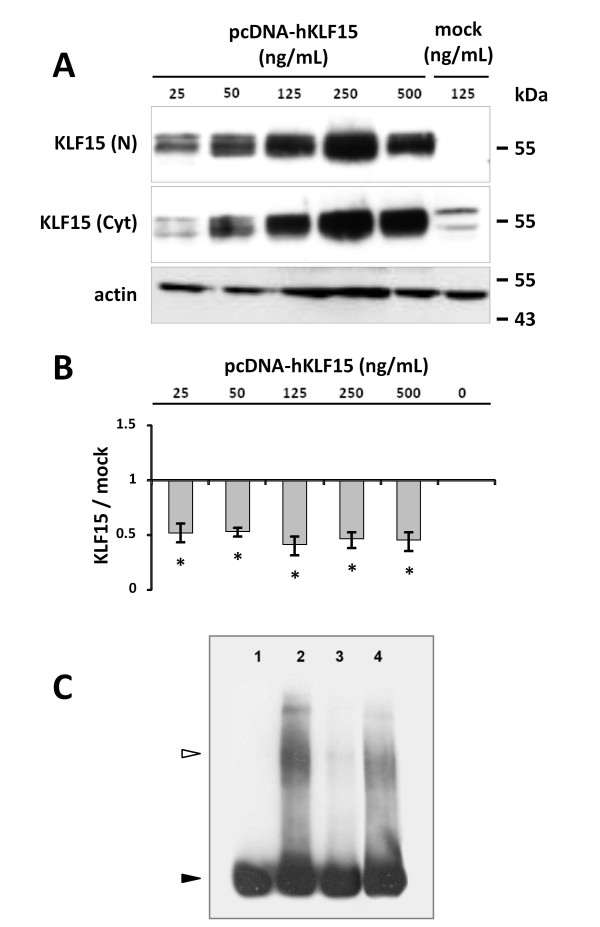
**Effect of KLF15 over-expression on P1 minimal promoter activity**. **(A) **Detection of KLF15 transcription factor in transiently transfected Rat2 cells. 48 hours after transfection of Rat2 cells with 25 to 500 ng/mL of pcDNA-hKLF15 or empty (mock) expression vector, nuclear (N) and cytoplasm (Cyt) proteins were extracted and 20 μg of total protein of each fraction were separated by SDS-PAGE. After transfer on nitrocellulose membrane, KLF15 was detected by chemiluminescence using anti-KLF15 antibody and horseradish peroxidase conjugated anti-goat IgG. Actin expression was used as a loading control. **(B) **Effect of KLF15 overexpression on *fut8 *P1 minimal promoter activity. Rat2 cells were co-transfected with 1 μg/mL of pGL3(-892/-451) vector containing the minimal promoter P1 sequence and 0 to 500 ng/mL of pcDNA-hKLF15 expression vector. 48 hours after transfection, *firefly *luciferase activity was measured and normalized by *Renilla *luciferase activity, as internal control. Histograms show a mean change in relative luciferase expression compared with control transfections, using the same concentration of empty vector (mock). ** P < 0.0005 (Student test). **(C) **EMSA analysis of nuclear proteins binding to (-892/-451) P1 promoter sequence. Lane 1, biotin 5'end-labeled (-892/-451) P1 promoter DNA probe; lane 2, labeled P1 promoter incubated with Rat2 cells nuclear proteins; lane 3, labeled P1 promoter incubated with Rat2 cells nuclear proteins in the presence of 200-fold molar excess of unlabelled sequence; lane 4, P1 promoter sequence incubated with nuclear proteins after pre-incubation with anti-KLF15 mAb. Back arrowhead indicates the electrophoretic mobility of biotin 5'end-labeled (-892/-451) P1 promoter DNA probe and open arrowhead the shift band obtained after incubation with Rat2 cells nuclear proteins.

In order to determine the effect of the potential repressor transcription factors on minimal promoter P1 activity, we co-transfected Rat2 cells with MZF1, PAX5, KLF15, IRF3 or PRDM1 expression vectors and with reporter vector pGL3(-892/-451) containing the minimal promoter P1 sequence. 48 hours after transfection, luciferase assays were performed and showed that only KLF15 was able to reduce the minimal promoter activity of T1 transcript by about 50%, at each concentration of vector tested (Figure 10B and additional file 1). These results were statistically significant (P < 0.0005) and suggested that KLF15 had repressor activity. A similar repressor effect of KLF15 was observed on the on pGL3(-1438/-451) construct (data not shown). The over-expression of KLF15 protein was checked by Western Blot in nucleus and cytoplasm fractions of Rat2 cells. As shown in Figure 10A, the anti-KLF15 mAb revealed two bands at about 55 kDa, in agreement with the apparent molecular weight of KLF15 protein in SDS-PAGE [32]. Endogenous KLF15 was mainly present in the cytosolic fraction (see mock cells) and the amount of KLF15 protein increased in both in nucleus and cytoplasm fractions with the concentration of transfected vector in a dose-dependent manner. At the lowest concentration of vector, KLF15 is mainly over-expressed in the nucleus fraction and the repression of luciferase activity for higher concentrations of vector doesn't change in spite of the increase of the protein in both fractions. These data indicate that the smallest quantity of KLF15 that accumulates in the nucleus is sufficient to observe a maximal repressor effect.

### Binding of nuclear proteins to P1 minimal promoter sequence

To investigate the association between nuclear proteins and the (-892/-451) P1 minimal promoter sequence, EMSA assay was performed to determine whether Rat2 cells nuclear proteins could bind this sequence. As shown in Figure 10C, a strong band was shifted (open arrow) when the nuclear extract was incubated with the biotinylated DNA fragment. The shifted band was significantly inhibited by a molar excess of unlabeled competitor. In order to further determine if KLF15 binds to the promoter sequence, super-shift assay was performed using anti-KLF15 mAb but no supplementary band was detected. This result indicates that KLF15 may not directly interact with the minimal promoter sequence.

## Discussion

The rat hybridoma cell line YB2/0 is considered as an alternative for the large-scale production of low fucose IgG [9]. The fucose transfer capacity of YB2/0 is much lower than the other rodent cell lines commonly used [27]. However, the amount of fucose-negative antibody produced by YB2/0 significantly decreased with the culture, due to the elevation of *fut8 *expression by the YB2/0 cells [27]. Important variations of the fucose content are also observed in production culture conditions.

To improve our knowledge of YB2/0 fucosylation machinery, we have cloned rat *fut8 *cDNA. *Fut8 *gene coding region spans over 140 kb of rat chromosome 6 and splits into nine coding exons with an organization closely related to that of the human ortholog [33]. The rat *fut8 *cDNA contains an ORF of 1725 bp, encoding a 575 amino acids polypeptide, showing 94% and 88% identity with the human and porcine enzymes, respectively. The amino acids sequence contains the three specific motifs of α1,6-fucosyltranferase in the catalytic domain (Figure 2). These motifs are shared by α2-fucosyltransferases, α6-fucosyltransferases, and protein-O-fucosyltransferases (POFUT), indicating that the corresponding genes have originated from a common ancestor by duplication and divergent evolution [34].

Interestingly, we identified 4 nucleotide changes in the coding sequence of rat *fut8 *cDNA when compared to the putative sequence (NM_001002289.1). Two of these SNP were also found in different EST sequences of the NCBI databases, showing the existence of a silent allelic polymorphism in rat gene, but without change in the amino-acid sequence of the polypeptide.

By FISH, we were able to determine the copy number of *fut8 *gene in YB2/0, showing a heterogeneous cell population, 60% of cells having three *fut8 *copies and 25.5% with 4 or more copies, the mean copy number being 2.8 ± 1.4 (Figure 3). Hybridoma cell lines such as YB2/0 are usually tetraploid but are also genetically instable. Depending on the culture medium, continuous cultivation induces chromosome rearrangements and loss of genetic material [35] that can explain *fut8 *heterogeneity. Even if other parameters dependent on the culture conditions may also influence *fut8 *expression during the cultivation, the heterogeneity of *fut8 *copy number should also have important consequences in fucosylation capacity of these cells.

RLM-RACE experiments with capped RNAs allowed to define for the first time the organization of the 5'-untranslated region of rat *fut8 *gene and to identify the different TSS. The rat *fut8 *gene 5'-untranslated region contains 4 exons spanning about 90 kb. Three mRNA isoforms were identified, arising from alternative splicing and alternative promoter usage, as shown in Figure 5. The quantification of T1 and T2 transcripts relative expression was performed by duplex Taqman™ QPCR, showing that T2 transcript was 1.6-fold higher expressed than the T1. This pattern of expression was similar in both YB2/0 and Rat2 cell lines. Several TSS were identified for each transcript, which is a common attribute for TATA-less promoter [36]. Data concerning *fut8 *5'-untranslated region and transcripts expression are rather limited. It has been shown that in the human SK-OV-3 ovarian cancer cells, *FUT8 *expression is controlled by the 5'-flanking region upstream exon 1. However, other 5'-untranslated sequences were found in ESTs, suggesting the presence of additional exons upstream exon 1, and that the transcription of the gene would be regulated by multiple promoters [37]. Two additional 5'-untranslated exons were identified upstream human *FUT8 *exon 1, which are expressed during embryogenesis as three groups of transcripts, suggesting an expression of *FUT8 *regulated by three different promoters [33]. From these data, it appears that rat and human *FUT8 *genes have a quite similar organization and pattern of expression, with at least three different transcripts encoding the same polypeptide.

The alternative first exons E-2 and E-3 are located about 90 kbp from the first coding exon E1 and separated by a 1 kbp intronic sequence. The 1 kbp rat genomic sequences upstream exons E-2 and E-3 were cloned in the pGL3b upstream the luciferase gene to determine the minimal promoter regions controlling the transcription of T1 and T2. These plasmids and 5'- or 3'-deleted constructions were transfected into YB2/0 and Rat2 cells for luciferase assays. However, the ratio of luminescence intensity for these different constructions compared to the control pGL3b was too low to allow determining the promoter activity in YB2/0. Nevertheless, we were able to delimitate the minimal promoter regions controlling the expression of both transcripts in Rat2 cells. We could show that the T1 transcript is controlled by a minimal promoter region within the sequence -892/-451 from ATG with two negative regulation regions within the -451/-411 and -1438/-1205 sequences. The binding of Rat2 cells nuclear proteins to the P1 minimal promoter region was confirmed by EMSA analysis. We also showed the existence of a minimal promoter region within the sequence -720/-537 upstream the ATG for transcript T2 and a strong positive regulation region in the exon E-3. Few are known about the promoters of *fut8 *in other species. Only one study has shown that in human ovarian cancer cells SK-OV-3, luciferase reporter assay indicated that the 1 kbp 5'-flanking region of exon E1 conferred the promoter activity. This region contains a TATA-box, but not a CCAAT motif, and potential binding sites for some transcription factors, such as bHLH, cMyb and GATA-1 [37].

Bioinformatics analysis of the promoter regions did not reveal any canonical TATA or CAAT boxes, but several putative binding sites for general transcription factors. Absence of TATA or CAAT boxes is a common feature of glycosyltransferase genes [38]. It is also in agreement with the presence of several TSS for the three transcripts, which is usual for such promoter regions. We also identified in both minimal promoter sequences several binding sites for transcription factors with repressor activity, which could be interesting targets to regulate *fut8 *in YB2/0. MZF1 (Myeloid Zinc Finger 1), PAX5 (Paired Box 5), KLF15 (Krüppel-like Factor 15), IRF3 (Interferon Regulatory Factor 3) and PRDM1 (Positive Regulatory Domain containing 1) consensus sequences have been identified in P1 promoter. MZF1 is known to regulate transcription of genes involved in cell growth, differentiation and apoptosis of myeloid cells. It is described as a repressor in non-hematopoietic cells and activator in hematopoietic cells [39]. PAX5 is known to down-regulate genes implicated in myeloid or T-lymphocyte differentiation in pro- B lymphocytes [40]. Interestingly, it has been shown that *fut8 *is up-regulated in PAX5-/- cells [40]. KLF15 has been demonstrated as a repressor of several important genes such the adrenomodullin gene in adipocytes [41] or the rhodopsin and the IRBP (Interphotoreceptor Retinoid Binding Protein) genes [42]. IRF3 is described as a repressor of RXRα, which is a key partner for number of nuclear receptors [43]. PRDM1 (alias Blimp1 or Prd1-Bf1) is known to repress several critical genes in B lymphocytes such as c-Myc [44]. HELT (Hey like transcriptional repressor), CDP (CCAAT displacement protein), MEL1 (MDS1/EVI1-like gene 1), Nkx3.1 (androgen-regulated homeobox protein), IKRS (Ikaros) and the zinc-finger transcription factor Gfi-1 were also identified in minimal promoter P2. HELT was shown to act as a transcriptional repressor in the neuronal development [45]. CDP is known to interact with regulatory element from a large number of genes involved in differentiation, cell growth and development. It is acting as a repressor by either a direct repressing effect when stably bound to the promoter, or by competing with activator factors for the occupancy of their binding sites [46]. MEL1 is described to have an essential function in the proliferation and maintenance of hematopoietic stem cells [47]. MEL1 is also known as a transcription repressor factor in the regulation of calreticulin expression, acting as a competitor with GATA6 factor [48]. Nkx3.1 is a homeodomain transcription factor, described as a transcriptional inhibitor of estrogen receptor activity, acting as a putative repressor in hormone-driven tumors [49]. IKRS factors are zinc finger DNA binding proteins. Six isoforms of IKAROS are described that often act as repressors. They seem to have a regulation role in the lymphocyte ontogeny [50]. Gfi-1 is described as a transcriptional repressor in lymphoid cell but its function can change with the cell context in hematopoietic development [51].

These transcriptional repressors can potentially bind to the *fut8 *minimal promoter regions and, in a first approach to control *fut8 *expression, the identified putative repressors of T1 transcript were co-transfected in Rat2 cells with reporter vector pGL3(-892/-451) containing the minimal promoter P1 sequence. No repressor activity was observed with MZF1, PAX5, IRF3 or PRDM1. In particular, we were not able to confirm the repressor activity of PAX5, as previously demonstrated in Pax5-/- cells [40]. However, KLF15 expression induced a 50% decrease of luciferase activity, whatever the concentration of vector. Western blot analysis of transfected cell lysates using anti-KLF15 mAb revealed two bands around 55 kDa. Whereas the theoretical molecular weight of KLF15 is about 45 kDa, this is in agreement with the apparent molecular weight of KLF15 protein previously observed in SDS-PAGE [32]. The 8 kDa difference between the two bands could be also explained by a post-translational modification such as SUMOylation, as it has been demonstrated for KLF5, another transcription factor of Krüppel Like Family [52]. Western blot showed that KLF15 is constitutively expressed in Rat2 cells, mainly in the cytosolic fraction, and increased in both nucleus and cytoplasm fractions with the concentration of transfected KLF15 expression vector in a dose-dependent manner. At the lowest concentration of vector, KLF15 is mainly over-expressed in the nucleus fraction. This is enough to observe a maximal repressor effect. This result indicates that a low expression of KLF15 in the nucleus results in a repression of the P1 minimal promoter activity.

## Conclusion

The rat hybridoma cell line YB2/0 is potentially an alternative for the large-scale production of low fucose IgG because of lower fucose transfer capacity compared to the other rodent cell lines commonly used. However, nothing was known about the rat α1,6-fucosyltransferase. Our results shed light on the *fut8 *gene organization and expression. Even if many other parameters such as GDP-Fuc concentration and transport or cell culture conditions can influence the fucosylation capacity of the cells, the heterogeneity of *fut8 *copy number in YB2/0 could explain at least in part the variation of fucose content in IgG producing cell populations and impedes efficient gene knockdown strategy. Alternatively, the presence of potential repressor binding sequences in the minimal promoter regions of *fut8 *could offer possible alternatives to control the fucosylation of IgG produced in these cells.

## Abbreviations

α1,6-FucT: the GDP-L-Fuc: N-acetyl-β-D-glucosaminide α1,6-fucosyltransferase; ADCC: Antibody-Dependent Cellular Cytotoxicity; CDP: CCAAT displacement protein; DMEM: Dulbecco's modified Eagle's medium; EMSA: electrophoretic mobility shift assays; EST: expressed sequence tags; FcγR: Fc fragment γ-receptor; FCS: fetal calf serum; FX: GDP-4-keto-6-deoxymannose 3,5-epimerase, 4-reductase; GFT: GDP-fucose transporter; GMD: GDP-mannose 4,6-dehydratase; HELT: Hey like transcriptional repressor; Ig: immunoglobulin; IKRS: Ikaros; IRF3: Interferon Regulatory Factor 3; KLF15: Krüppel-like Factor 15; mAb: monoclonal antibody; MEL1: MDS1/EVI1-like gene 1; MZF1: Myeloid Zinc Finger 1; NKX3.1: androgen-regulated homeobox protein; NRP: non relevant probe; PAX5: Paired Box 5; pGL3b: pGL3-basic vector; PRDM1: Positive Regulatory Domain containing 1; QPCR: quantitative real-time polymerase chain reaction; RLM-RACE: RNA-Ligase-Mediated Rapid Amplification of cDNA Ends; TSS: transcription start site.

## Authors' contributions

BT carried out the molecular cloning, the enzyme activity characterization, transcripts' analysis and partially drafted the manuscript. EM carried out the P2 promoter characterization, MB carried out the P1 promoter characterization. AHL has performed the bioinformatic analysis. CG, AF & SJ participated in the design and coordination of the study. AF performed FISH analysis and partially drafted the manuscript. PhD conceived of the study, participated in its design and coordination and drafted the manuscript. All authors read and approved the final manuscript.

## Supplementary Material

Additional file 1 **Effect of transcription factors over-expression on *fut8 *P1 minimal promoter activity**. Bioinformatics analysis identified consensus binding sites for PRDM1, IRF3, PAX5, MZF1 and KLF15 putative repressors in the T1 transcript minimal promoter sequence. Human coding sequence of PRDM1, IRF3 and MZF1 were cloned into the pcDNA3.1 expression vector using the Gateway^® ^conversion system (Invitrogen, Carlsbad, CA, USA). The pcDNA3.1 vector containing the human KLF15 sequence was kindly provided by Dr. Otteson (College of Optometry, University of Houston, TX, USA) and the pcDNA3.1 vector containing the human sequence of PAX5 was provided by Dr. Broccardo (Centre de Physiopathologie INSERM U563, Toulouse, France). Rat2 cells were co-transfected with 1 μg/mL of pGL3(-892/-451) vector containing the minimal promoter P1 sequence and 0 to 750 ng/mL of PRDM1 (A), IRF3 (B), PAX5 (C), MZF1 (D) or KLF15 (E) expression vector. 48 hours after transfection, *firefly *luciferase activity was measured and normalized by *Renilla *luciferase activity, as internal control. Histograms show a mean change in relative luciferase expression compared with control transfections, using the same concentration of empty vector (mock). * P < 0.01, ** P < 0.0005 (Student test). Click here for file
