# Supplementary figures and images for: Molecular cloning, characterization, genomic organization and promoter analysis of the α1,6-fucosyltransferase gene (fut8) expressed in the rat hybridoma cell line YB2/0
Source: BMC Biotechnol. 2011 Jan 5;11:1. doi: 10.1186/1472-6750-11-1 (PMC3022693; doi:10.1186/1472-6750-11-1)

## Slide 1
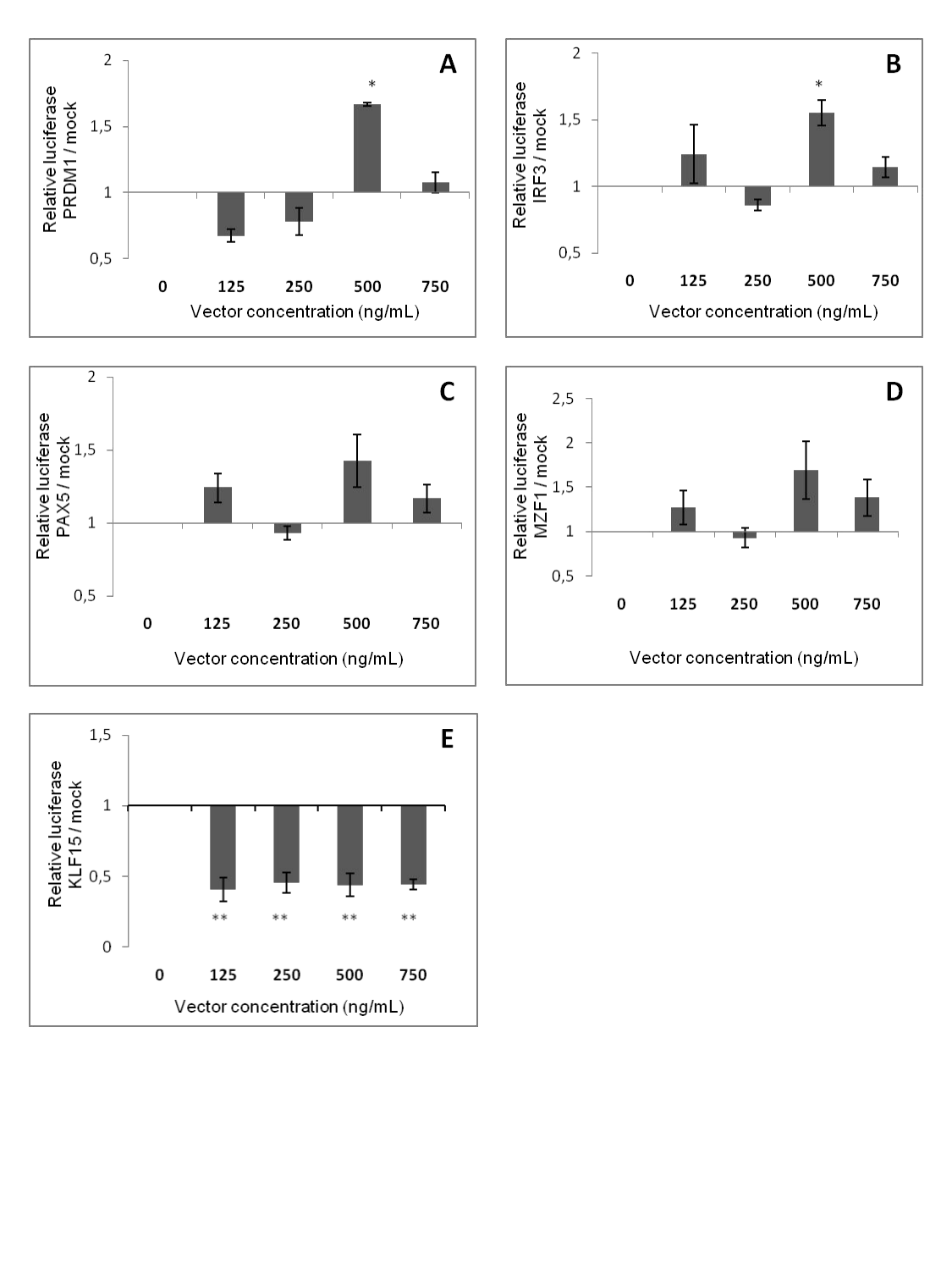

Supplement: Additional file 1 — Effect of transcription factors over-expression on fut8 P1 minimal promoter activity. Bioinformatics analysis identified consensus binding sites for PRDM1, IRF3, PAX5, MZF1 and KLF15 putative repressors in the T1 transcript minimal promoter sequence. Human coding sequence of PRDM1, IRF3 and MZF1 were cloned into the pcDNA3.1 expression vector using the Gateway® conversion system (Invitrogen, Carlsbad, CA, USA). The pcDNA3.1 vector containing the human KLF15 sequence was kindly provided by Dr. Otteson (College of Optometry, University of Houston, TX, USA) and the pcDNA3.1 vector containing the human sequence of PAX5 was provided by Dr. Broccardo (Centre de Physiopathologie INSERM U563, Toulouse, France). Rat2 cells were co-transfected with 1 μg/mL of pGL3(-892/-451) vector containing the minimal promoter P1 sequence and 0 to 750 ng/mL of PRDM1 (A), IRF3 (B), PAX5 (C), MZF1 (D) or KLF15 (E) expression vector. 48 hours after transfection, firefly luciferase activity was measured and normalized by Renilla luciferase activity, as internal control. Histograms show a mean change in relative luciferase expression compared with control transfections, using the same concentration of empty vector (mock). * P < 0.01, ** P < 0.0005 (Student test). [file 1472-6750-11-1-S1.PPT]
